# PEALD-Grown Crystalline AlN Films on Si (100) with Sharp Interface and Good Uniformity

**DOI:** 10.1186/s11671-017-2049-1

**Published:** 2017-04-18

**Authors:** Sanjie Liu, Mingzeng Peng, Caixia Hou, Yingfeng He, Meiling Li, Xinhe Zheng

**Affiliations:** School of Mathematics and Physics, Beijing Key Laboratory for Magneto-Photoelectrical Composite and Interface Science, University of Science and Technology Beijing, No. 30, Xueyuan Road, Beijing, China

**Keywords:** Aluminum nitride, PEALD, Sharp interface, Good uniformity

## Abstract

Aluminum nitride (AlN) thin films were deposited on Si (100) substrates by using plasma-enhanced atomic layer deposition method (PEALD). Optimal PEALD parameters for AlN deposition were investigated. Under saturated deposition conditions, the clearly resolved fringes are observed from X-ray reflectivity (XRR) measurements, showing the perfectly smooth interface between the AlN film and Si (100). It is consistent with high-resolution image of the sharp interface analyzed by transmission electron microscope (TEM). The highly uniform thickness throughout the 2-inch size AlN film with blue covered surface was determined by spectroscopic ellipsometry (SE). Grazing incident X-ray diffraction (GIXRD) patterns indicate that the AlN films are polycrystalline with wurtzite structure and have a tendency to form (002) preferential orientation with increasing of the thickness. The obtained AlN films could open up a new approach of research in the use of AlN as the template to support gallium nitride (GaN) growth on silicon substrates.

## Background

With a direct wide bandgap of 6.2 eV [1], high resistivity and resistance of breakdown voltage, and good thermal conductivity and stability [2], aluminum nitride (AlN) is suitable for various applications, such as photodetectors, ultraviolet light-emitting diodes, complementary metal-oxide-semiconductor (CMOS), and solar cells. As we know, low-temperature prepared AlN was used as a critical buffer layer for the growth of epitaxial gallium nitride (GaN) layers on sapphire substrates [3–5], which contributed to the development of GaN electronic and optoelectronic devices. Since large and high-quality silicon wafers are readily available at relatively low cost, AlN films grown on silicon substrates are highly desirable and have the potential to develop GaN electronic and optoelectronic devices on silicon substrates in future. Recently, ultrathin AlN films deposited at low temperatures were widely applied for passivation layers on high electron mobility transistors (HEMTs) by controlling their thickness at atomic level [6–11]. Therefore, great efforts have been carried out for fabricating high-quality AlN growth at low temperature. It is well known that plasma-enhanced atomic layer deposition (PEALD) is a low-temperature growth method based on self-limiting growth mechanism, which can deposit highly uniform and conformal angstrom-scale thin films. In the literatures, Alevli et al. [12] fabricated polycrystalline AlN films using PEALD, and the polar (002)-preferred orientation appeared with increasing the temperature up to 400 °C. Ozgit et al. [13] obtained (100)-oriented polycrystalline AlN films on Si (100) substrates. Epitaxial growth of (002)-oriented crystalline AlN films on GaN and sapphire were achieved [14, 15]. However, high-quality (002)-preferred orientation AlN films on silicon substrates have not been realized at low temperature up to now.

In this work, we have deposited polycrystalline hexagonal AlN films with (002) preferential orientation on Si (100) substrates at temperature as low as 300 °C. Interface between the AlN film and Si (100) has been investigated. AlN films with sharp interface and good uniformity are obtained.

## Methods

AlN thin films were deposited on Si (100) substrates using an Angstrom-dep III PEALD reactor, Thin Film Technologies Ltd. of USA, equipped with an inductively coupled remote plasma (ICP) source. After cleaned by RCA standard cleaning process, the substrates are immediately loaded onto the reactor chuck in the PEALD system and pumped down to the system base pressure of ~0.15 Torr. Once reaching the system base pressure, the substrate chuck is heated resistively to the growth temperature. Upon at deposition temperature, another 20 min is needed for temperature to reach a balance. After that, the substrate surfaces are treated with high-purity (HP) Ar/N_2_/H_2_ (1:3:6) plasma to form NH groups, followed by purging the chamber with HP Ar. Subsequently, HP trimethylaluminum (TMA) is introduced separately for AlN growth. Unreacted TMA and by-products are removed by purging the chamber with HP Ar. Thus, each cycle of AlN growth consists of Ar/N_2_/H_2_ plasma pulse/Ar purge/TMA pulse/Ar purge. Table 1 summarizes the deposition conditions of the AlN films by PEALD.

**Table 1:** Deposition conditions of the AlN films by PEALD

Precursor 1	TMA (99.999%)
Precursor 2	Ar/N_2_/H_2_ (99.999%)
Carrier gas	Ar (99.999%)
Gas line temperature	60 °C
Flow rate of carrier gas (UHP Ar)	5 sccm
Flow rate of N precursor	5 sccm
RF power	60 W
RF plasma frequency	13.56 MHz

After deposition, the thickness and the optical constants of AlN films were measured by spectroscopic ellipsometer (SE) in the energy range of 1.5–4.5 eV at incidence angle of 70°. X-ray reflectivity (XRR) with a PANalytical system X-ray reflectometry was used to study the interface between the films and substrates. The crystallinity of the as-deposited AlN was analyzed by grazing incidence X-ray diffraction (GIXRD) measurement. The thickness, uniformity, and interface of the as-deposited AlN films were further characterized by transmission electron microscope (TEM).

## Results and Discussion

**Fig. 1:**
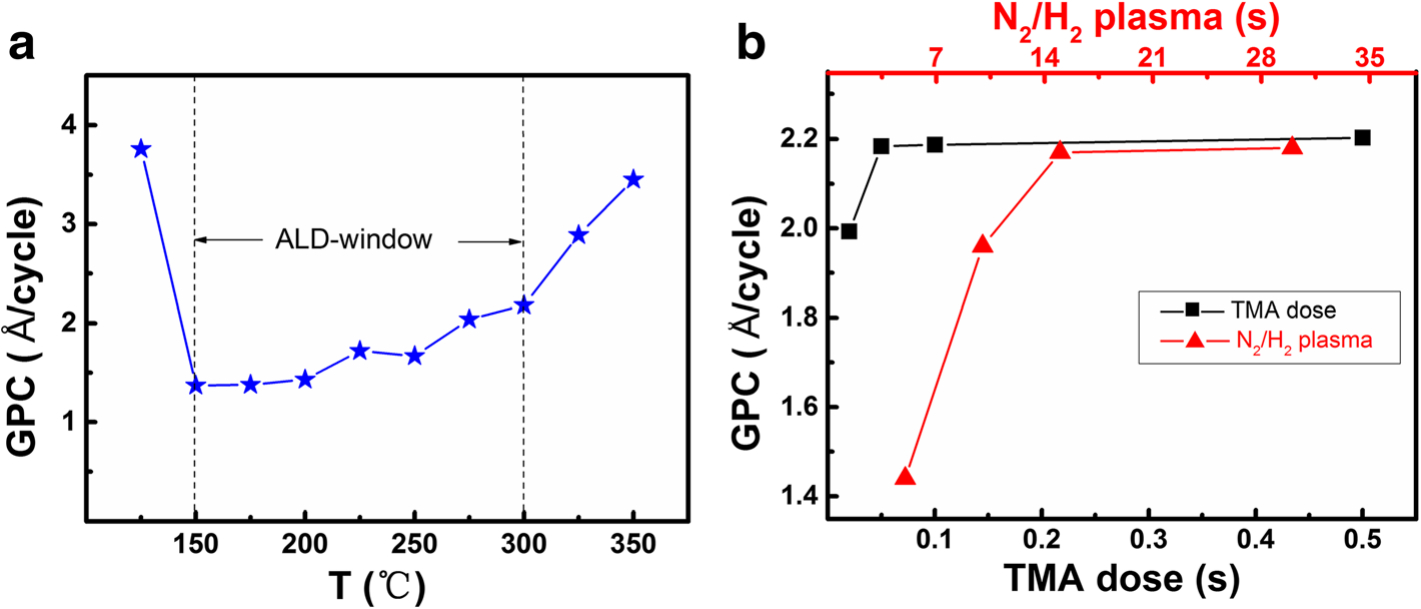
**a** Deposition rate of AlN thin films on Si (100) at different temperature. **b** Precursor saturation curves at 300 °C. *Black square* represents N_2_/H_2_ plasma that was kept constant at 30 s for TMA dose saturation curves. *Black triangle* represents N_2_/H_2_ plasma saturation curve with an optimal TMA dose of 0.05 s

Deposition rate, also called growth per cycle (GPC), as a function of temperature is shown in Fig. 1a. It is obvious that GPC decreases from 3.76 to 1.4 Å/cycle with the temperature increases from 125 to 150 °C due to the condensation of TMA. The value of GPC within the temperature range of 150–300 °C is lower than the literal value of 2.5 Å/cycle [16], indicating a self-limited growth process. When the deposition temperature is above 300 °C, the GPC increases with the increase of growth temperature due to the self-decomposition of TMA molecules [17]. Hence, the temperature range of 150–300 °C is atomic layer deposition window for this case, where the GPC varies with the deposition temperature. This variation can be attributed to the effect of temperature on the number and type of reactive sites present on the surface [18]. Figure 1b shows the saturation curves of aluminum and nitrogen precursors obtained at 300 °C. For the TMA saturation curve, GPC increases with TMA dose until 0.05 s, where the deposition rate saturates at ~2.18 Å/cycle. For the N_2_/H_2_ plasma saturation curve, GPC increases with increased N_2_/H_2_ plasma pulse time until a constant deposition rate is obtained at 15 s. The achieved deposition rate of ~2.18 Å/cycle is higher than the reported values of PEALD-grown AlN [19–25] owing to the component of our plasma Ar:H_2_:N_2_ (1:6:3) in this work. The H_2_:N_2_ ratio is sufficient for AlN deposition process [22] in terms of the required N source. On the other hand, Ar acts as an ignition providing plasma with enough energy to overcome steric hindrance of methyl groups and make more complete reducing reaction.

**Fig. 2:**
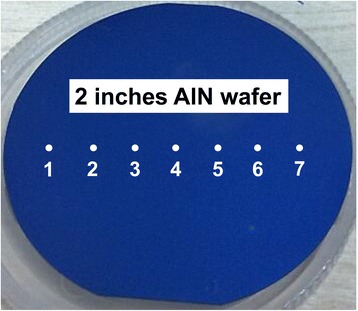
Schematic points for the thickness measurement of AlN/Si (100)

Based on the optimized PEALD parameters, the AlN film was deposited on the 2-inch size Si (100) substrate at 300 °C for 400 cycles. For this as-deposited AlN film, no apparent interference fringes are observed under naked eyes, implying the good uniformity in thickness through the entire wafer. To verify the good uniformity of this sample, seven schematic points in Fig. 2 were measured by SE. The average thickness of the film is 86.6 nm figured out by using the results in Table 2. via the equation

**Table 2:** The extracted thickness of the 2-inch size AlN film at different points

Thickness/nm	86.1	86.5	86.8	86.7	87.0	86.8	86.5
Points	1	2	3	4	5	6	7

$$ \eta =\frac{d\left( \max \right)- d\left( \min \right)}{2 d\left(\mathrm{average}\right)}, $$

where *η* and *d* are the non-uniformity and thickness of the film, respectively, the non-uniformity *η* of around 1% is calculated, suggesting that the AlN nucleation on Si (100) is highly uniform. Deposition of large-size uniform AlN films by PEALD at low temperatures broadens application of AlN in the areas that require uniform growth at low temperature with thickness controlled at the atomic level.

**Fig. 3:**
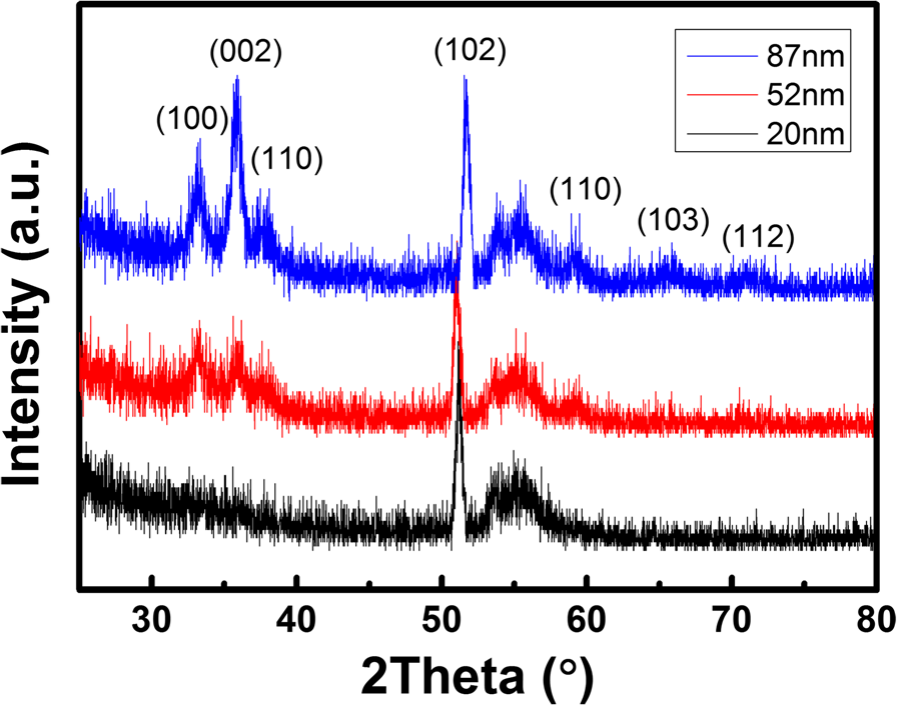
GIXRD patterns for different thick AlN films on Si (100) at 300 °C

Figure 3 shows the X-ray diffraction XRD patterns of AlN thin films deposited at 300 °C with different thickness. From XRD analysis, it can be observed that AlN layers are polycrystalline. For the 87-nm-thick AlN film, different diffraction peaks at 2*θ* value of 33.2°, 35.8°, 37.7°, 51.7°, 59.3°, 65.0°, and 71.0° are assigned to the (100), (002), (101), (102), (110), (103), and (112) planes of hexagonal AlN, respectively, based on the PDF card (no: 01-080-6097). AlN films display a higher degree of crystallization with increasing of thickness. For the 20-nm-thick AlN, only the (102) peak is evident. When the thickness is increased to 52 nm, peaks for (100), (002), (101), (102), and (110) planes can be found in the XRD patterns. The (002) preferential orientation appears as the thickness increases to 87 nm, indicating that AlN films promote crystallization in (002) plane with increasing of thickness. Consequently, epitaxial growth of (002)-oriented AlN is feasible by improving the film thickness to a certain value. As we know, raising the process temperature is conductive to the formation of single-crystalline structure. Hence, increasing both of the thickness of AlN films and deposition temperature of PEALD process might suppress the polycrystalline nature of the film and form a single-crystalline AlN by PEALD on silicon substrate, and we will make further efforts to study about it in the future.

**Fig. 4:**
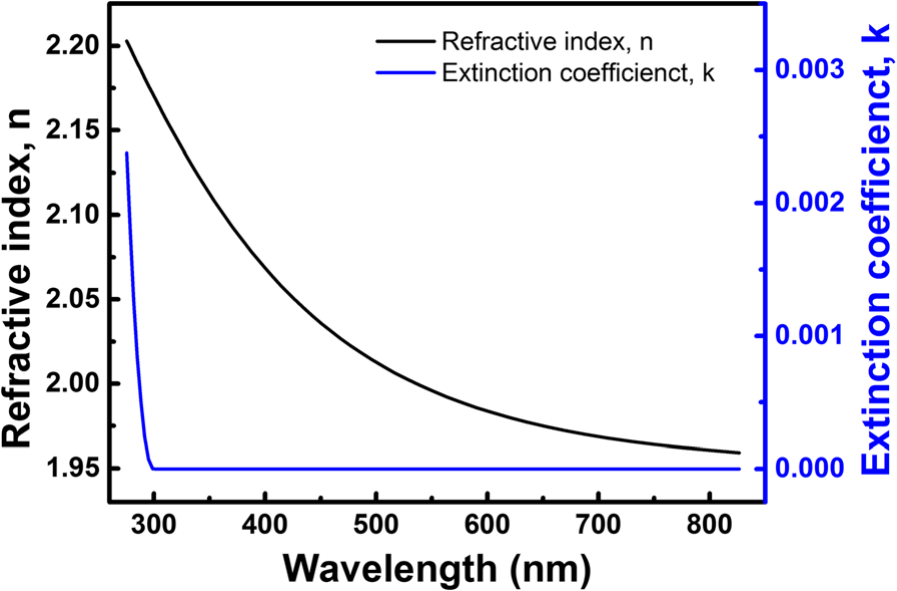
Optical constants (refractive index and extinction coefficient) of the 87-nm AlN thin film

The optical performance of the 87-nm-thick AlN film is analyzed in Fig. 4. Refractive index (*n*) decreases from 2.20 to 1.96 with increasing wavelength from 275 to 826 nm. The value of *n* at 632 nm is 1.97, which is consistent with the value reported by Barshilia et al. [26]. The extinction coefficient (*k*), which is 0.002 at 275 nm, decreases rapidly within the wavelength range of 275–300 nm. For higher wavelengths *k* is zero, indicating that films are transparent above 300 nm in wavelength, which is a red shift as compared with the band-edge wavelength of single-crystalline AlN. This feature might widen the utilization of AlN films as a window in solar photovoltaic technology.

**Fig. 5:**
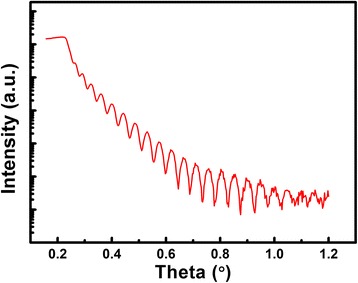
XRR measurement of the 87-nm AlN thin film

**Fig. 6:**
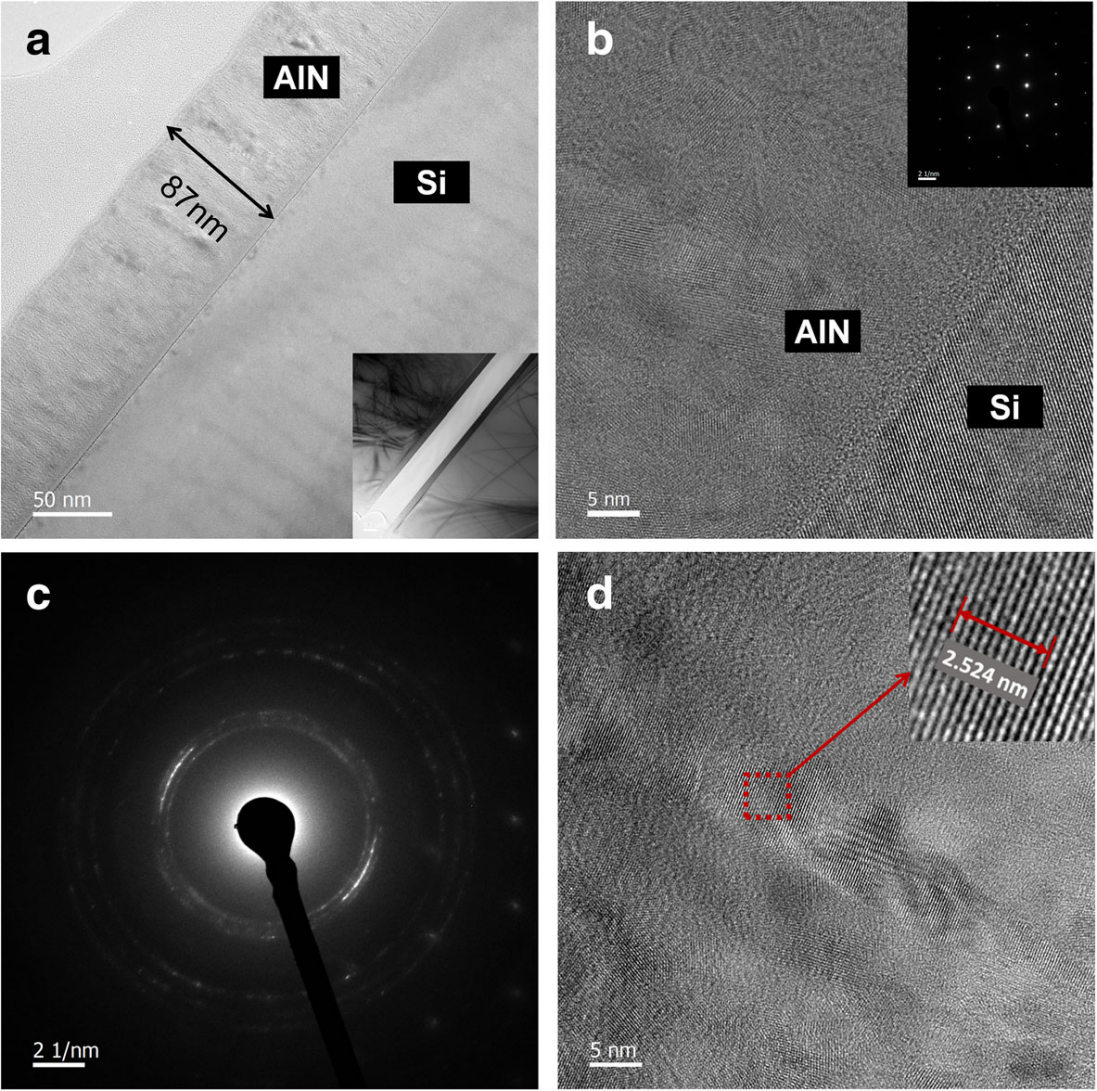
Cross-sectional TEM images of the 87-nm AlN thin film. **a** Cross-sectional TEM image and (*inset*) lower magnification cross-sectional TEM image. **b** Cross-sectional HR-TEM image of the same sample and (*inset*) SAED pattern of Si (100) substrate. **c** SAED pattern of the same sample. **d** Cross-sectional HR-TEM image and (*inset*) magnification of the selected square area

It is well known that the interface between AlN passivation layer and III-nitride surface is crucial for the reduction of the current collapse in AlGaN/GaN HEMTs [7]. The clearly resolved fringes from XRR measurement in Fig. 5 reveal that the interface between the AlN film and Si (100) is perfectly smooth. The film thickness of around 87 nm can be obtained using a fit to the experimental curve (not shown here), which is in good agreement with the thickness measured by TEM as shown in Fig. 6a. Inset of Fig. 6a shows that the film is highly uniform, verifying the good uniformity measured by SE. Figure 6b is the high-resolution TEM (HR-TEM) image of the same sample. The sharp interface is observed between the as-deposited AlN and Si (100) consistent with the results of XRR as shown in Fig. 5. This image also shows that the AlN is amorphous at the initial thickness of ~5 nm and then starts to crystallize. A hexagonal lattice structure is shown in the corresponding selected-area electron diffraction (SAED) pattern (Fig. 6c), which confirms the results made by GIXRD. With the increase of film thickness, AlN gradually exhibits a preferred orientation, as presented in Fig. 6d. Inset of Fig. 6d is a magnification of the square area, the distance for ten lattice planes of which is measured as 2.524 nm, corresponding to the (002) plane of hexagonal AlN materials. From both XRR measurements and HR-TEM images, we can conclude that the AlN films deposited on Si (100) substrates have sharp interface. This feature of sharp interface between as-deposited AlN and Si (100) could benefit for the deposition of the passivation layer or the window layer as a conformal way.

## Conclusions

In summary, polycrystalline hexagonal AlN films with sharp interface and good uniformity have been deposited on Si (100) at 300 °C by PEALD. Increasing the thickness of AlN films promotes crystallization in (002) orientation. AlN films exhibit a high transparency in the visible region of the spectrum, which can be utilized in solar photovoltaic technology. The achieved AlN films are not only potential buffer layer materials for GaN growth but also promising materials for applications in other microelectronic and optoelectronic devices.

## References

[1] Yamashita H, Fukui K, Misawa S, Yoshida S (1979). Optical properties of AlN epitaxial thin films in the vacuum ultraviolet region. J Appl Phys.

[2] Junior AF, Shanafield DJ (2004). Thermal conductivity of polycrystalline aluminum nitride (AlN) ceramics. Cerâmica.

[3] Akasaki I, Amano H, Koide Y, Hiramatsu K, Sawaki N (1989). Effects of AlN buffer layer on crystallographic structure and on electrical and optical properties of GaN and Ga1−xAlxN (0<x ≦ 0.4) films grown on sapphire substrate by MOVPE. J Cryst Growth.

[4] Hiramatsu K, Itoh S, Amano H, Akasaki I, Kuwano N, Shiraishi T, Oki K (1991). Growth mechanism of GaN grown on sapphire with AlN buffer layer by MOVPE. J Cryst Growth.

[5] Nakamura S (1991). In situ monitoring of GaN growth using interference effects. Jpn J Appl Phys.

[6] Koehler AD, Nepal N, Anderson TJ, Tadjer MJ, Hobart KD, Eddy CR, Kub FJ (2013). Atomic layer epitaxy AlN for enhanced AlGaN/GaN HEMT passivation. Electron Device Letters, IEEE.

[7] Huang S, Jiang Q, Yang S, Zhou C, Chen KJ (2012). Effective passivation of AlGaN/GaN HEMTs by ALD-grown AlN thin film. Electron Device Letters, IEEE.

[8] Huang S, Jiang Q, Yang S, Tang Z, Chen KJ (2013). Mechanism of PEALD-grown AlN passivation for AlGaN/GaN HEMTs: compensation of interface traps by polarization charges. Electron Device Letters, IEEE.

[9] Tang ZK, Huang S, Jiang Q, Liu SG, Liu C, Chen KJ (2013). High-voltage (600-V) low-leakage low-current-collapse AlGaN/GaN HEMTs with AlN/SiN_x_ passivation. Electron Device Letters, IEEE.

[10] Liu XY, Zhao SX, Zhang LQ, Huang HF, Shi JS, Zhang CM, Lu HL, Wang PF, Zhang DW (2015). AlGaN/GaN MISHEMTs with AlN gate dielectric grown by thermal ALD technique. Nanoscale Res Lett.

[11] Chen KJ, Huang S (2013). AlN passivation by plasma-enhanced atomic layer deposition for GaN-based power switches and power amplifiers. Semicond Sci Technol.

[12] Alevli M, Ozgit C, Donmez I, Biyikli N (2012). Structural properties of AlN films deposited by plasma-enhanced atomic layer deposition at different growth temperatures. Phys Status Solidi A.

[13] Ozgit C, Donmez I, Alevli M, Biyikli N (2012). Self-limiting low-temperature growth of crystalline AlN thin films by plasma-enhanced atomic layer deposition. Thin Solid Films.

[14] Nepal N, Qadri SB, Hite JK, Mahadik NA, Mastro MA, Eddy CR (2013). Epitaxial growth of AlN films via plasma-assisted atomic layer epitaxy. Appl Phys Lett.

[15] Tarala V, Ambartsumov M, Altakhov A, Martens V, Shevchenko M (2016). Growing c-axis oriented aluminum nitride films by plasma-enhanced atomic layer deposition at low temperatures. J Cryst Growth.

[16] Danielsson Q, Janzen E (2003). Using N_2_ as precursor gas in III-nitride CVD growth. J Cryst Growth.

[17] Riihela D, Ritala M, Matero R, Leskela M, Jokinen J, Haussalo P (1996). Low temperature deposition of AIN films by an alternate supply of trimethyl aluminum and ammonia. Cherc Vap Deposition.

[18] Puurunen RL (2005). Surface chemistry of atomic layer deposition: a case study for the trimethylaluminum/water process. J Appl Phys.

[19] Kim KH, Kwak NW, Lee SH (2009). Fabrication and properties of AlN film on GaN substrate by using remote plasma atomic layer deposition method. Electron Mater Let.

[20] Alevli M, Ozgit C, Donmez I, Biyikli N (2011). The influence of N_2_/H_2_ and ammonia N source materials on optical and structural properties of AlN films grown by plasma enhanced atomic layer deposition. J Cryst Growth.

[21] Bui HV, Wiggers FB, Gupta A, Nguyen MD, Aarnink AAI, Jong MP, Kovalgin AY (2015). Initial growth, refractive index, and crystallinity of thermal and plasma-enhanced atomic layer deposition AlN films. J Vac Sci Technol.

[22] Goerkea S, Zieglera M, Ihringa A, Dellitha J, Undiszb A, Diegela M, Andersa S, Huebnera U, Rettenmayrb M, Meyer HG (2015). Atomic layer deposition of AlN for thin membranes using trimethylaluminum and H_2_/N_2_ plasma. Appl Surf Sci.

[23] Ozgit C, Goldenberg E, Okyay AK, Biyikli N (2014). Hollow cathode plasma-assisted atomic layer deposition of crystalline AlN, GaN and Al_x_Ga_1−X_N thin films at low temperatures. J Mater Chem C.

[24] Bosund M, Sajavaara T, Laitinen M, Huhtio T, Putkonen M, Airaksinen VM, Lipsanen H (2011). Properties of AlN grown by plasma enhanced atomic layer deposition. Appl Surf Sci.

[25] Motamedi P, Cadien K (2015). Structural and optical characterization of low-temperature ALD crystalline AlN. J Cryst Growth.

[26] Barshilia HC, Deepthi B, Rajam KS (2008). Growth and characterization of aluminum nitride coatings prepared by pulsed-direct current reactive unbalanced magnetron sputtering. Thin Solid Films.

